# Nematic Structures under Conical Anchoring at Various Director Tilt Angles Specified by Polymethacrylate Compositions

**DOI:** 10.3390/polym13172993

**Published:** 2021-09-03

**Authors:** Denis A. Kostikov, Mikhail N. Krakhalev, Oxana O. Prishchepa, Victor Ya. Zyryanov

**Affiliations:** 1 Kirensky Institute of Physics, Federal Research Center KSC SB RAS, 660036 Krasnoyarsk, Russia; kmn@iph.krasn.ru (M.N.K.); p_oksana@iph.krasn.ru (O.O.P.); zyr@iph.krasn.ru (V.Y.Z.); 2 Institute of Engineering Physics and Radio Electronics, Siberian Federal University, 660041 Krasnoyarsk, Russia

**Keywords:** conical anchoring, polymethacrylate, nematic liquid crystal, orientational structure, director tilt angle, domain wall, electrically-induced reorientation

## Abstract

Dependence of the director tilt angle of nematic liquid crystal (LC) under conical anchoring from the two-component polymer mixture composition has been studied. We varied the ratio of poly(isobutyl methacrylate) (PiBMA), which specifies a conical anchoring for the nematic liquid crystal LN-396, and poly(methylmethacrylate) (PMMA) assigning a tangential alignment for the same nematic. An oblique incidence light technique to determine a tilt angle has been used. It has been shown that the tilt angle increases from $0^{\circ }$ to $47.7^{\circ }$ when PiBMA:PMMA ratio changes in the range 30:70 to 100:0. The specific optical textures viewed under the polarizing microscope and proper orientational structures have been considered for various compositions of the polymer films. An electric field action on the formed orientational structures has been investigated. The obtained results are promising for the application in various electro-optical LC devices with a conical anchoring in which the director tilt angle is a crucial parameter: a controlled diffraction gratings, an electrically operated achromatic rotators of linear light polarization, etc.

## 1. Introduction

**Figure 1:**
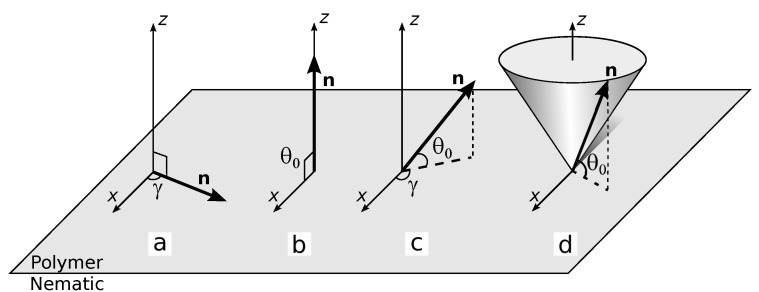
Different types of boundary conditions at the nematic-polymer interface: (**a**) tangential anchoring with specified azimuthal angle γ, (**b**) homeotropic anchoring, (**c**) tilted anchoring with specified azimuthal angle γ and tilt angle $\theta_{0}$, (**d**) conical anchoring with specified tilt angle $\theta_{0}$ and azimuthal degeneration.

Liquid crystals (LC) have the long-range order of molecules that determines the anisotropy of their properties [1]. Optical, electric, magnetic, and other LC characteristics depend on the director field distribution **n** (the unit vector directed along the preferred orientation of LC molecules), which, in turn, is determined by boundary conditions, material LC parameters, and a cell size [2]. Boundary conditions assign both an initial LC orientation and its response to the external influences [3]. Nowadays, LC structures under tangential ($\theta_{0}$ angle between the director and substrate plane is $0^{\circ }$) (Figure 1a) or homeotropic ($\theta_{0}≅90^{\circ }$) (Figure 1b) boundary conditions are widely applied in the displays, electrically controlled wave plates, polarization rotators, phase spatial light modulators, and et al. It should be noted that usually the boundary conditions with strictly $\theta_{0}=0^{\circ }$ or $\theta_{0}=90^{\circ }$ angle are not used in such devices, and the director has a pre-tilt angle (about a few degrees). It allows to avoid forming a random domain structure and obtain a definite and repeatable structure response to an electric field applied perpendicular to the substrates.

Specifying anchoring conditions that differ from the tangential or homeotropic ones can result in complex structures with unique or improved electro-optical characteristics and the realization of new orientational transitions. For example, electrically-induced switching between twisted and untwisted nematic structures occurs under tilted boundary conditions ($0<\theta_{0}<90^{\circ }$) (Figure 1c) [4]. The electro-controllable lenses [5,6,7] and the phase gratings [8,9,10,11], the chiral nematic structure with the tilted homogeneous orientation of helicoid axis [12], Q-plates with different topological characteristics [13], and other superstructures of cholesteric [14] are created by the assignment of the specific azimuthal and polar director orientation on the substrates. There are some methods to assign the required boundary conditions. Azimuthal director orientation can be specified by using the photo-sensitive polymers or dyes for which a polymerization process or molecules orientation depends on the initiating radiation polarization [15]. The director tilt angle can be controlled by selecting the type and concentration of surfactants [16], polymers, their mixtures, or processing technology. For instance, the tilt angle varies in the wide range $0\leq \theta_{0}\leq 90^{\circ }$ by the mixture composition of two polymers specifying homeotropic and tangential boundary conditions [17,18,19,20,21], depth and time of rubbing of orienting polymer films [18,19,22,23,24], freezing of the formed director orientation using photo-cured polymer dissolved in LC [20,22,25,26,27,28,29], the time and temperature of annealing of polyamide film [19,21,24,30], etc. In most cases, when assigning the director tilt angle, the homogeneous azimuthal orientation is formed due to the rubbing of both LC cell substrates.

LC systems with conical boundary conditions are rarer. In this case, the tilt angle is in the range $0<\theta_{0}<90^{\circ }$, but there is an azimuthal degeneration of the director orientation (Figure 1d). Such boundary conditions are formed at the interface between LC and its own isotropic phase [31,32] or LC and isotropic liquid doped with surfactant [33,34,35,36]. For LC 5CB a conical anchoring with tilt angles $\theta_{0}=18^{\circ }$ and $\theta_{0}=16^{\circ }$ is specified on the solid substrates covered with the polystyrene or polyisoprene, respectively [37]. Poly(perfluoro(4-vinyloxy-1-butene)) (CYTOP) specifies conical boundary conditions with tilt angle depending on temperature for LC [30,38]. Poly(isobutyl methacrylate) assigns conical boundary conditions with tilt angle $\theta_{0}=50^{\circ }$ for the nematic mixture LN-396 [39,40].

Because of the azimuthal degeneration of director orientation under conical anchoring, various orientational structures without additional surface treatment can be obtained. For example, in the nematic LC cell under tangential boundary conditions on the bottom substrate and conical anchoring on the top one (tangential-conical anchoring), a domain structure with a uniform azimuthal director orientation is formed [40]. In this case, the azimuthal degeneration of conical anchoring is eliminated by rubbed polymer film on the bottom substrate. The structure of strips characterized by the periodically-varied azimuthal director orientation is formed in chiral nematic under tangential-conical anchoring when the ratio value of the layer thickness *d* to the cholesteric pitch *p* is larger than the critical one [41]. At that, the line orientation and, consequently, azimuthal director orientation on the substrate with conical anchoring can be operated by varying the LC layer thickness [42] or applying an electric field [43]. At a lower d/p value, the twisted nematic-like structure is formed [40], and the azimuthal director angle on the substrate with conical anchoring can be controlled by an electric field applied perpendicularly to the substrates [44]. It allows obtaining the electrically controllable achromatic rotator of light polarization [45]. The value of tilt angle at conical anchoring determines the LC cell thickness *d* at which such a light polarization rotator can be realized. Nowadays, there are no data concerning the possibility of assigning the director tilt angle at conical anchoring in the wide range of angles.

In the present work, we study the dependence of the director tilt angle, the formed orientational structures of nematic, and their response to an electric field from the polymers mixture composition. We used the polymer mixture with variable ratio of two polymethacrylates, one of which specifies tangential boundary conditions for nematic, and the other specifies the conical ones.

## 2. Materials and Methods

Sandwich-like LC cells based on the glass substrates with ITO layer coated with polymer layers have been studied. The mixture poly(isobutyl methacrylate) (PiBMA) (Sigma Aldrich, St. Louis, MO, USA) and poly(methyl methacrylate) (PMMA) (Sigma Aldrich, St. Louis, MO, USA) with various weight ratios or the mixture PiBMA:PMMA doped with nematic LN-396 (Belarusian State Technological University) have been used as orienting polymer films. Polyvinyl alcohol (PVA) (Sigma Aldrich) has been used as tangentially orienting film. The polymer films were deposited on the substrates by spin coating. Then, 1% PVA water solution, and 1% solution of PiBMA:PMMA mixture or PiBMA:PMMA doped with LC in butyl acetate were used. Next, the films were dried for 1 h at 90 ${}^{\circ }$C temperature. After drying the PVA film was unidirectionally rubbed. The films based on the polymer mixture PiBMA:PMMA were either rubbed or used without additional treatment. Three LC cell types have been studied: (*i*) both substrates were covered with the rubbed films of PiBMA:PMMA mixture; (*ii*) one substrate was covered with the rubbed PVA film and the other was covered with the unrubbed film of PiBMA:PMMA mixture; (*iii*) one substrate was covered with the rubbed PVA film and the other was covered with the unrubbed film of PiBMA:PMMA mixture doped with 20% LN-396. LC cells were filled with the nematic mixture LN-396 (Belarusian State Technological University) by the capillary method. LN-396 consists of 40% 4-n-pentyl-4${}^{\prime }$-cyanobiphenyl (5CB), 20% 4${}^{\prime }$-propoxybiphenyl-4-carbonitrile (3OCB), 15% 4${}^{\prime }$-butoxy-biphenyl-4-carbonitrile (4OCB), 18% 4-butyl-cyclohexanecarboxylic acid 4-ethoxy-phenyl ester (4CEPO2), and 7% 4-butylcyclohexanecarboxylic acid 3${}^{\prime }$-methyl-4${}^{\prime }$-pentyl-biphenyl ester (by weight). The melting temperature of LN-396 is $T_{m}=-20{\;}^{\circ }$C, the clearing point is $T_{c}=+66{\;}^{\circ }$C, anisotropy of dielectric conductivity is Δε=10.2. LC layer thickness was assigned by the glass microspheres (Duke Scientific, Fremont, CA, USA) of 9.6±1.0 μm diameter (LC cells of type *(i)*) or 17.3±1.4 μm diameter (LC cells of type *(ii)* and *(iii)*).

**Figure 2:**
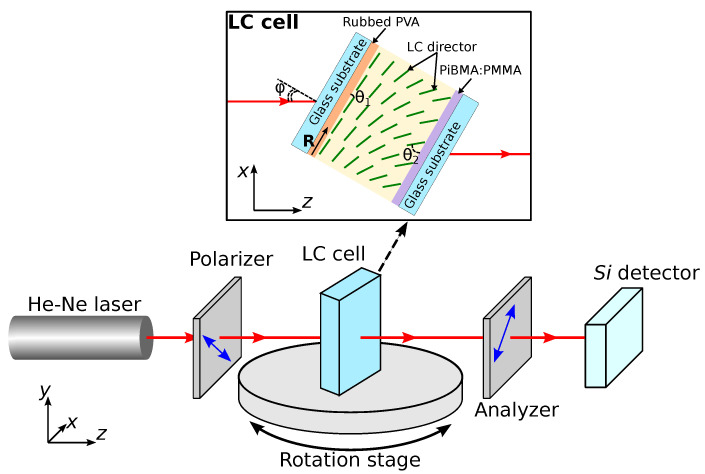
Scheme of optical setup to measure the director tilt angle on substrates by the oblique light incidence method. The inset is the scheme of LC cell.

Formed LC structures and their electrically-induced transformations have been examined by the polarizing optical microscopy (POM) with Axio Imager.A1m (Zeiss, Jena, Germany) microscope. 1 kHz AC voltage of varied amplitude was applied perpendicular to LC layer. The dependence of light transmittance of the sample placed between the crossed polarizers from the incidence angle φ was used to measure the director tilt angle at the substrates. The measurements were carried out with the optical setup (see Figure 2). He-Ne laser beam (λ=632.8 nm) passes sequentially through the polarizer, LC cell, analyzer, and falls into the photo-detector. The sample was rotated about the *y*-axis (in xOz-plane), and the rubbing direction **R** of the substrate with PVA film was in xOz-plane (**R** is parallel to the *x*-axis at the normal incidence of the laser beam). The light transmittance was measured in the range of incidence angle $-50^{\circ }\leq \varphi \leq 50^{\circ }$ with the step of $1^{\circ }$. The polarizer and analyzer directions were at the angles of $-45^{\circ }$ and $45^{\circ }$ to the *x*-axis, respectively.

The director tilt angles on substrates were found by matching the experimental transmittance curve and theoretical dependence obtained from the phase delay calculated in the approximation of linear variation of the director tilt over the layer thickness. The phase delay is calculated as [46]:

(1) $$\Gamma \left(\varphi \right)=\int_{\theta_{1}}^{\theta_{2}}\frac{2\pi d}{\lambda (\theta_{2}-\theta_{1})}\left[\frac{(n_{⊥}^{2}-n_{\|}^{2})\sin\theta \cos\theta }{n^{2}}\sin\varphi +\frac{n_{⊥}n_{\|}\sqrt{n^{2}-\sin^{2}\varphi }}{n^{2}}-\sqrt{n_{⊥}^{2}-\sin^{2}\varphi }\right]\;d\theta ,$$

where $\theta_{1}$ and $\theta_{2}$ are the tilt director angles at the substrates, *d* is LC layer thickness, φ is the light incidence angle, $n^{2}=n_{⊥}^{2}\cos^{2}\theta +n_{\|}^{2}\sin^{2}\theta$. $n_{\|}=1.720$ and $n_{⊥}=1.520$ are the refractive indices for light at the wavelength λ=632.8 nm polarized parallel and perpendicular to the director, correspondingly. The transmittance of the LC cell in crossed polarizers is:

(2) $$T_{calc}\left(\varphi \right)=\sin^{2}\left[\frac{\Gamma (\varphi )}{2}\right].$$

The experimental transmission of LC cell is found as $T_{exp}\left(\varphi \right)=I\left(\varphi \right)/I_{max}$, where I(φ) is the passed light intensity, $I_{max}$ is the maximal value of I(φ). Theoretical curves $T_{calc}\left(\varphi \right)$ calculated at different combinations of tilt angles and cell thickness were matched to the experimental data by the least-squares method to find the director tilt angles at the substrates. The calculations and matching of data were carried out in the programming language GNU Octave.

## 3. Results and Discussion

### 3.1. Orientational Structures

#### 3.1.1. LC Cells Based on the Substrates with Rubbed PiBMA:PMMA Films

**Figure 3:**
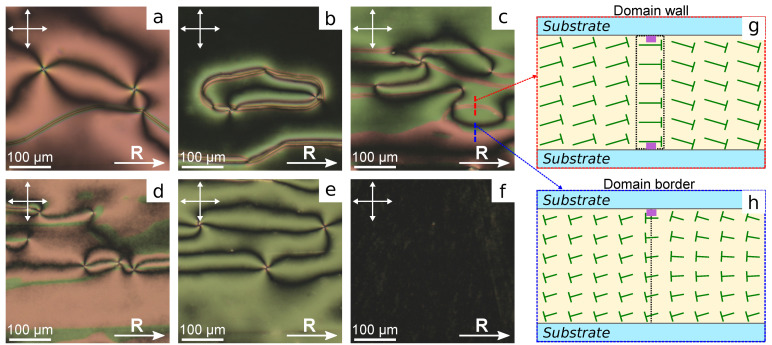
POM photos of LC cells with substrates covered with PiBMA:PMMA mixture with weight ratio polymers equal to: (**a**) 100:0, (**b**) 80:20, (**c**) 60:40, (**d**) 40:60, (**e**) 20:80, and (**f**) 0:100. Scheme of the director orientation in LC cell cross-section along (**g**) red and (**h**) blue dash lines. Nails show the director orientation oblique to the figure plane. The nail length is proportional to the director projection onto the figure plane. Acute ends of nails correspond to the director pointing towards the observer. Hereinafter, the solid arrow indicates the rubbing direction **R**, the polarizer directions are indicated by double arrows.

Polymer PiBMA specifies a conical anchoring with the azimuthal degeneration of director on the substrate for nematic LN-396. When PiBMA films are unidirectionally rubbed (ratio PiBMA:PMMA = 100:0), the azimuthal degeneration of director orientation is also in the LC cell (Figure 3a). Consequently, the structure with point surface defects-boojums and domain Bloch’s walls where the boundary conditions get broken to tangential is formed in the sample.

A homogeneous structure of director oriented along the rubbing direction **R** arises in the LC cell with rubbed PMMA films (ratio PiBMA:PMMA = 0:100) (Figure 3f). In the sample with the polymer ratio PiBMA:PMMA = 80:20 the preferred orientation of director along the rubbing direction is formed (Figure 3b). At that, the Bloch’s domain walls and some number of boojums located, as a rule, near the walls are observed. Another situation realizes in the samples with lower content of PiBMA polymer in the orienting films. For example, there is no preferred director orientation in the sample with the polymer ratio PiBMA:PMMA = 60:40 (Figure 3c). There are domain walls and a significant amount of boojums, as well as defects with semi-numerical topological charge. The domain walls separate the sample areas with opposite signs of the director’s tilt angles on both substrates (Figure 3g). Generally, the director is not parallel to the domain wall in its center, in contrast to the Bloch walls formed at higher concentrations of PiBMA in orienting films. In addition, the domains with different colors are observed in the sample under crossed polarizers. Since the color of the interference pattern depends on the phase delay between ordinary and extraordinary waves, these color domains differ in the sign of the director tilt angle on the top and bottom substrates (Figure 3h). The same sign of director tilt angle on substrates corresponds to the lesser phase delay, and when the director tilt angles on the top and bottom substrates have the opposite signs, the phase delay is greater. At the domain’s border the sign of director tilt angle changes only on one of the substrates (Figure 3h). It should be noted that the domain walls and domain borders are oriented preferably along the rubbing direction. A similar structure is observed in the LC cell with PiBMA:PMMA = 60:40 mixture as orienting layers (Figure 3d). In this case, a lesser number of domain walls are formed, but the domain quantity of different colors is greater. Further increase in polymer PMMA part in orienting layers results in structures without domains and walls (Figure 3e). It means that PiBMA:PMMA = 20:80 mixture assigns a small director tilt angle at the substrates. At that, the inhomogeneous director orientation with the point surface defects is formed in the sample, in contrast to the cell with the pure PMMA as an orienting layer.

The appearance of domains in LC cells at the definite polymer ratios PiBMA:PMMA indicates that the director tilt angle on the substrate is not zero. However, the inhomogeneity of director orientation does not allow determining the director tilt angle at substrates. At that, the director orientation at the conical boundary conditions is susceptible to the orienting effect of the substrate with planar anchoring when the substrates with various boundary conditions are used [40]. In this case, the nematic structure with homogeneous azimuthal director orientation along the rubbing direction must form.

#### 3.1.2. LC Cells Based on Substrates with Rubbed PVA Film and Non-Rubbed PiBMA:PMMA Film

**Figure 4:**
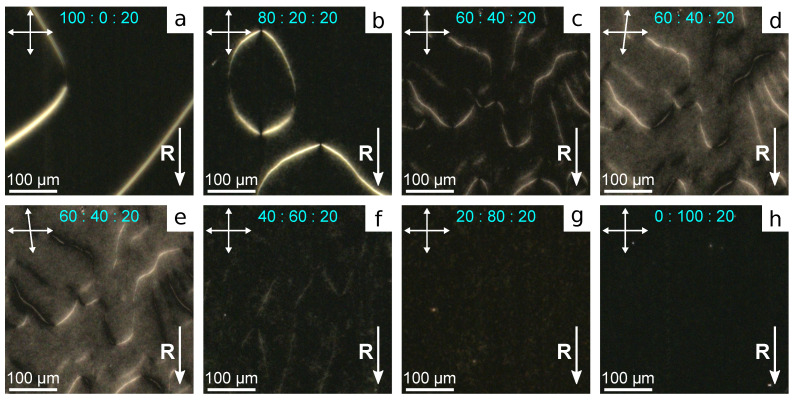
POM photos of LC cells with the bottom substrate covered with the rubbed PVA film and the top substrate covered with the film of polymer and LC mixture with the weight ratio PiBMA:PMMA:LN-396 equal to: (**a**) 100:0:20, (**b**) 80:20:20, (**c**–**e**) 60:40:20, (**f**) 40:60:20, (**g**) 20:80:20, and (**h**) 0:100:20. Photos are taken (**a**–**c**,**f**–**h**) in crossed polarizers, and at the angle between analyzer and polarizer (**d**) $83^{\circ }$ and (**e**)$-83^{\circ }$.

When one substrate is covered with the rubbed PVA film, and another one is covered with the non-rubbed film of PiBMA:PMMA mixture, the structure with almost homogeneous azimuthal director orientation is formed in LC cells. Since the transition temperature of polymer PiBMA ($T_{g}=65{\;}^{\circ }$C) is close to the clearing point of LN-396 ($T_{c}=66{\;}^{\circ }$C), LC cells have been filled with nematic at room temperature (in mesophase). Then, for the several samples, including LC cell with pure PMMA, the structure with the non-homogeneous azimuthal director orientation with the twist deformation is formed, which is apparently caused by flow effects (see Supplementary Materials Figure S1). This effect can be eliminated by adding 20% LC LN-396 into the polymer mixture. Such an amount of LN-396 is dissolved entirely, particularly, in polymer PiBMA [47], and LC droplets are not formed in PiBMA: PMMA film. In this case the structure with homogeneous azimuthal director orientation along the rubbing direction of PVA film is formed in all the samples (Figure 4).

The domain structure with opposite director tilt angle proper to the system with tangential-conical anchoring [40] is formed in the samples with polymer ratio PiBMA: PMMA:LN-396 in the range from 100:0:20 to 40:60:20 inclusive (Figure 4a–f). Varying boundary conditions to tangential ones accompanied by twist deformation of the director field is observed at the domain border. At the substrate with conical anchoring, the director is oriented parallel to the domain border. Consequently, the domain border is maximal dark when the analyzer is parallel to it and the polarizer is perpendicular to the rubbing direction **R** at the bottom substrate (Figure 4d,e). The domain border contains the reversing points separating the areas with opposite signs of the director twist (azimuthal director angle), whose quantity is always an even number when the domain border is closed. The average domain size grows with the increase in polymer PiBMA part in the orienting film. It is evidently due to the increase in director tilt angle. In this case, the surface energy related to the broken boundary conditions increases, which results in a decrease in all the lengths of the domain borders. The twist deformation of the director field and reversing points on the domain borders assign a response and relaxation of the structure on the electric field.

### 3.2. An Electric Field Effect on the Orientational Structures

**Figure 5:**
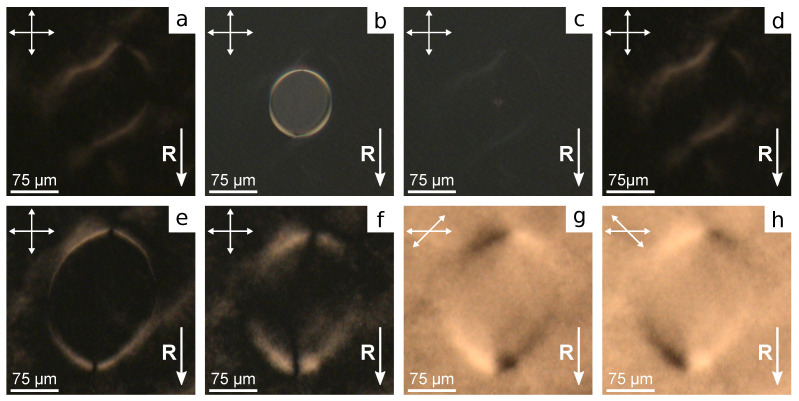
POM photos of two areas of LC cell with the bottom substrate covered with rubbed PVA film and the top substrate covered with a film of the polymer and LC mixture with weight ratio PiBMA:PMMA:LN-396 = 60:40:20. The first area (**a**) before, (**b**) in 40 s, (**c**) 80 s after voltage-on U=6.0 V, and (**d**) after voltage-off. Photo of the second area (**e**) before and (**f**–**h**) after switching-off voltage U=3.0 V. Photos are taken (**a**–**f**) in crossed polarizers, and at the angle between analyzer and polarizer (**g**) $45^{\circ }$ and (**h**)$-45^{\circ }$.

Rubbing PVA film makes the director pre-tilt angle, that is $\theta_{1}=0.6^{\circ }\pm 0.3^{\circ }$ for nematic LN-396. Therefore, the domains with the different signs of director tilt angle are not equivalent, which conditions their stability under external factors, for example, an electric field. Nematic mixture LN-396 has a positive anisotropy of permittivity and, hence, the director **n** tends to orient along to the applied field. The director reorientation under the applied voltage decreases the domain size with the opposite sign of director tilt angles at the top and bottom substrates (Figure 5a–c). The decrease in the domain size is caused by a movement and gradual shrinking of their borders. The dynamics of this process as well as relaxation of the structure after voltage-off depend on the polymer ratio PiBMA:PMMA and the value of the applied field.

For the LC cell with the film of PiBMA:PMMA:LN-396 = 100:0:20 the domains shrink under the applied voltage starting from the threshold voltage $U_{th}≅4.0$ V. After the complete contraction and disappearance of domains, a homogeneous azimuthal orientation of the director is observed. Adding PMMA into the orienting film changes the response of the structure on an electric field. Firstly, the threshold voltage decreases with the increase in the PMMA part in the film, and it is $U_{th}≅2.0$ V for the mixture PiBMA:PMMA:LN-396 = 40:60:20. Secondly, the domain shrink rate grows as voltage increases. Thirdly, if the domain border stays in one location long enough, then here the azimuthal director orientation proper to the domain border is remembered.

For instance, Figure 5a demonstrates the area with domain in LC cell with PiBMA:PMMA:LN-396 = 40:60:20 film before applied voltage. The domain size along the horizontal side of the picture is about 175μm. At U=6.0 V the domain shrinks and collapses for 80 s (Figure 5b,c). After the voltage is off a “trace” with the azimuthal distribution of director field identical to the one before the electric field action is observed in the site of the initial location of domain border (Figure 5d). This “trace” gradually disappears for 40 min, and a homogeneous azimuthal director’s orientation is formed.

If the rate of domain shrinking decreases, then a temporary memorizing of the director’s azimuthal orientation occurs through the whole surface where the domain border moves. For example, the applied voltage U=3.0 V, that is slightly above the threshold value, leads to the shrinking and collapse of the domain for 30 min. (Figure 5e,f). In this case, azimuthal deviation of the director from rubbing direction **R** occurs not only at the site of the initial location of domain border but inside the domain’s area. At that, the angle of director deviation from R is greatest near the initial domain borders, and it gradually decreases towards the domain center, which can be seen both in crossed polarizers and at analyzer turning (Figure 5f–h).

**Figure 6:**
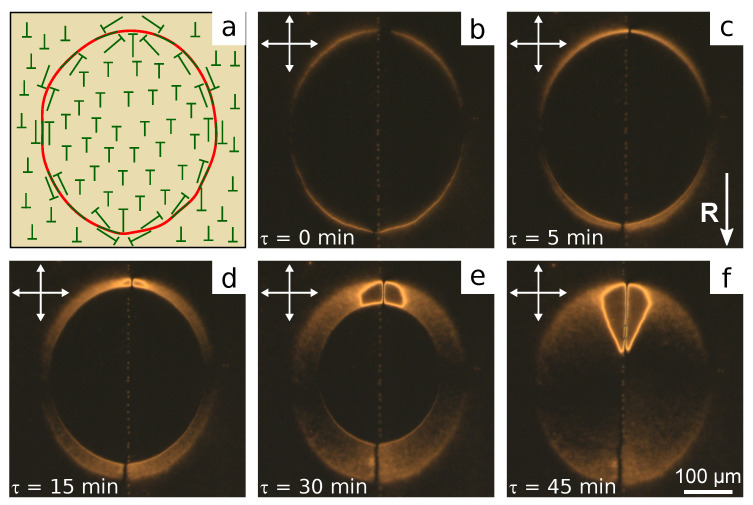
(**a**) Scheme of the director orientation at the substrate covered with PiBMA:PMMA:LN-396 = 80:20:20 mixture before the voltage action. The position of the domain border is indicated by the red line. POM photos of the sample area with the domain (**b**) before and after the action of voltage U=10 V during τ equal to: (**c**) 5 min, (**d**) 15 min, (**e**) 30 min, and (**f**) 45 min.

**Figure 7:**
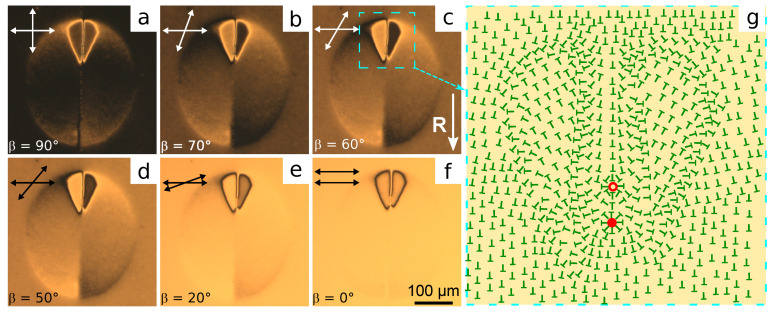
POM photos of the sample area with substrate covered with PiBMA:PMMA: LN-396 = 80:20:20 mixture after collapsing of the domain under the action of voltage U=10 V are taken at the angle between the analyzer and polarizer β equal to: (**a**) $90^{\circ }$, (**b**) $70^{\circ }$, (**c**) $60^{\circ }$, (**d**) $50^{\circ }$, (**e**) $20^{\circ }$, and (**f**) $0^{\circ }$. (**g**) The scheme of director orientation in the area with a significant deformation. The boojums with topological charges −1 and +1 are indicated by the red unpainted and painted circles, respectively.

The increase in the PiBMA part in the orienting film results in the growth of the domain sizes, the increase in the threshold field, and the decrease in the domain shrinking rate. Accordingly, the temporary memorizing of the azimuthal director orientation caused by the movement of domain border is revealed more distinctly. Moreover, the configurations with the azimuthal director turn by more than $90^{\circ }$ can appear in the process of response and relaxation of structures. Figure 6 shows the domain in the LC cell with the orienting film of PiBMA:PMMA:LN-396 = 80:20:20. Initially, the domain is an ellipse with a pair of reversing points located at the major axis. Under U=10.0 V, the domain size reduces slowly, and its shape keeps Figure 6c–e. One can see that the movement of the domain border located close to one of the reversing points (the top point in Figure 6) leads to the formation of significant director deformations. As a rule, such deformations appear near the reversing point for which the motion direction forms an acute angle with the **R** vector. In this area, the maximal rotation angle of the director is $\pm 180^{\circ }$, which is seen using the rotating analyzer method (Figure 7).

Rotating analyzer method has shown that the structure formed after shrinking of the domain is mirror-symmetric to the plane formed by the major axis of domain ellipse and the normal to the substrates. The azimuthal turning of the director agrees with the director’s behavior near the domain border in most of the area. That is, the director turns clockwise in the bottom right and top left parts of the area previously occupied by the domain. Conversely, the director turns anticlockwise in the bottom left and top right parts of the collapsed domain. Inside the area with a significant deformation, the director turns approximately $\pm 155^{\circ }$ anticlockwise to the right of the symmetry plane (the dark area in Figure 7b,c) and clockwise to its left. The transition from the inner to the outer area is accompanied by a sharp change of the director orientation (sharp bright or dark lines in Figure 7a or Figure 7f). At the same time, two point features (defects) from which a pair of extinction bands appear get seen. The extinction bands near one defect turn in the same direction as the analyzer, while they turn on the opposite side for the second defect. It means that point defects are two boojums with topological charges +1 and −1, respectively. The scheme of corresponding director orientation at the substrate covered with PiBMA:PMMA:LN-396 mixture is shown in Figure 7g.

The structures with significant deformations of the director can be formed at the domain shrinking in the samples with the orienting film of PiBMA:PMMA:LN-396 = 70:30:20, 80:20:20, 90:10:20. In all the cases, the temporary structures resulted from after the electric field action are characterized by azimuthal deviation of director orientation from the rubbing direction only. The tilt angle of the director at the substrates does not change, which can be seen in crossed circular polarizers. In this case, the interference pattern depends only on the phase delay between ordinary and extraordinary waves (see Supplementary Figure S2). The complex configuration formed after the shrinking of the domain relaxes into a state with the homogeneous azimuthal orientation of the director over time. Thus, the structure with homogeneous azimuthal orientation and the equal sign of polar tilt angle of the director can be obtained for all the studied samples either initially (at low PiBMA concentration) or after the applying of voltage. It allowed us to measure the director tilt angles at the substrates covered with the orienting films with different compositions using the oblique light incidence method.

### 3.3. A Tilt Angle

**Figure 8:**
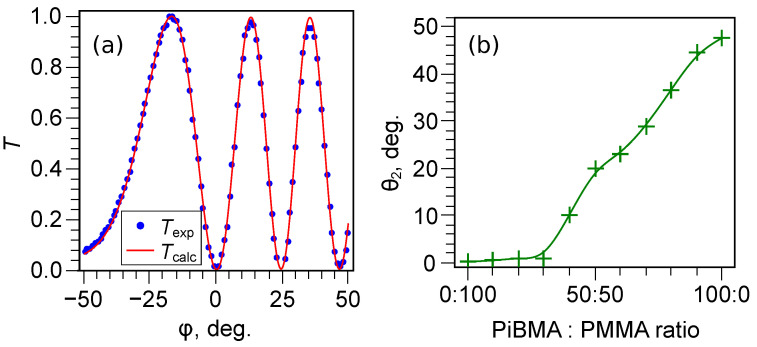
(**a**) Experimental $T_{exp}$ and theoretical $T_{calc}$ dependencies of transmittance from the incidence light angle φ obtained for LC cell with the orienting film of PiBMA:PMMA:LN-396 = 70:30:20. The theoretical dependence $T_{calc}$ is obtained for an LC cell with the thickness d=20.5μm, and the director tilt angles $\theta_{1}=0.3^{\circ }$ and $\theta_{2}=28.9^{\circ }$. (**b**) Dependence of the director tilt angle $\theta_{2}$ at the substrate covered with the PiBMA:PMMA:LN-396 mixture from the polymer ratio.

Due to the non-zero values of the director tilt angles at the substrates with tangential or conical anchoring, the dependence of the transmittance T(φ) from the incident angle of light is not symmetric to $\varphi =0^{\circ }$ (Figure 8a). This makes it possible to determine unambiguously the director tilt angles at substrates $\theta_{1}$ and $\theta_{2}$ and LC layer thickness *d*. For all the samples under study, the director tilt angle at the rubbed PVA film is $\theta_{1}=0.6^{\circ }\pm 0.3^{\circ }$. The obtained dependence of the director tilt angle $\theta_{2}$ from the composition of the orienting film of PiBMA:PMMA:LN-396 is shown in Figure 8b. One can see that the director tilt angle at the PMMA orienting film (PiBMA:PMMA:LN-396 = 0:100:20) is $\theta_{2}≅0^{\circ }$ (tangential boundary conditions). Similar $\theta_{2}$ values are obtained when the part of PiBMA reaches the ratio PiBMA:PMMA:LN-396 = 30:70:20. Further PiBMA fraction increase leads to an almost linear growth of the director tilt angle $\theta_{2}$, which reaches the value $\theta_{2}=47.7^{\circ }\pm 0.6^{\circ }$ for the PiBMA film (PiBMA:PMMA:LN-396 = 100:0:20).

The director tilt angle is $\theta_{2}≅0^{\circ }$ at the low fraction of polymer PiBMA in the orienting film, and, therefore, the domain structure in LC cells does not arise. The forming of a relatively small director tilt angle, such as $\theta_{2}=10.1^{\circ }\pm 0.4^{\circ }$ at the ratio PiBMA:PMMA:LN-396 = 40:60:20, results in the domain structure in LC cells both with the same orienting films at the substrates (Figure 3d) and under tangential boundary conditions at one of the substrates (Figure 4f). The measured value $\theta_{2}=47.7^{\circ }\pm 0.6^{\circ }$ for the PiBMA film (without polymer PMMA) is in good agreement with the value of the director tilt angle $\theta {}_{0}=50^{\circ }\pm 4^{\circ }$ obtained for the droplets of nematic LN-396 dispersed in PiBMA [39].

The flow effect disturbing the homogeneous azimuthal orientation of the director is absent in the LC cell when one substrate is covered with rubbed PVA film, and the other is covered with only PiBMA (PiBMA:PMMA:LN-396 = 100:0:0). It allowed us to measure the director tilt angle at the polymer film PiBMA without nematic LN-396. The obtained value $\theta_{2}=49.8^{\circ }\pm 0.6^{\circ }$ is practically equal to the director tilt angle at the PiBMA film doped with LN-396. Thus, we can conclude that 20% LC added into the polymer mixture does not affect the tilt angle $\theta_{2}$. In this case LC is a plasticizer for the used polymer mixture [48] and increases the mobility of polymer chains. This, in turn, provides forming boundary conditions with an azimuthal degeneration of the director orientation (conical anchoring) [37]. This is revealed in the elimination of the flow effects and in the absence of long-term memorization of the azimuthal orientation of the director, which is characteristic of domain boundaries. Since conical boundary conditions are observed for a pure PiBMA film (undoped with LN-396), then, mainly increasing mobility of PMMA chains is necessary. In particular, in ref. [49] the surface gliding effect on the aligning PMMA film was obtained by heating the LC cell above the transition temperature of PMMA.

## 4. Conclusions

Orientational structures of nematic LN-396 formed in LC cells with orienting films of the mixtures PiBMA:PMMA or PiBMA:PMMA:LN-396 have been studied. The homogeneous director orientation is formed in LC cell at the rubbed films with the ratio of PiBMA:PMMA = 0:100 only. Other compositions under study show the azimuthal degeneration of director orientation. An increase in the fraction of PiBMA to PiBMA:PMMA = 40:60 and more results in the domain structure. When one substrate is covered with the rubbed PVA film, and the other is covered with a non-rubbed film of PiBMA:PMMA mixture, the structure with the almost homogeneous azimuthal orientation of the director is formed in LC cells. At that, the azimuthal deviation of director orientation caused by the flow effect is observed in the number of samples. This effect is eliminated by adding 20% nematic LN-396 into the polymer PiBMA:PMMA mixture. In such a case, the homogeneous structure is formed at the ratio PiBMA:PMMA:LN-396 in the range from 0:100:20 to 30:70:20. At a higher concentration of PiBMA in the mixture, the domain structure arises.

Under the electric field applied perpendicular to LC layer, the domains shrink and disappear. The shrinking rate depends on the value of applied voltage and the ratio of polymers in the orienting film. If the domain border on which the boundary conditions get broken moves slowly, then the director azimuthal orientation deviates from the rubbing direction and is temporarily memorized. At the sufficiently high content of PiBMA polymer in the mixture, the domain shrinking can result in structures with significant deformations of the director field. These structures disappear over time, and the homogeneous defect-less director configuration is formed.

The director tilt angles at the orienting films with different polymer proportions have been measured. PMMA film specifies the tangential anchoring ($\theta_{2}≅0^{\circ }$). For a PiBMA fraction in the mixture more than PiBMA:PMMA:LN-396 = 30:70:20, the director tilt angle grows with the increase in PiBMA part, reaching $\theta_{2}=47.7^{\circ }\pm 0.6^{\circ }$ for PiBMA:PMMA:LN-396 = 100:0:20 mixture. The formation of the homogeneous azimuthal orientation of the director in LC cells with planar anchoring at one substrate (a rubbed PVA film) allows us to conclude that the films of PiBMA:PMMA:LN-396 specify conical boundary conditions with tilt angle depending on the ratio of the polymer PiBMA:PMMA. The obtained results are promising for the design of various electro-optical systems based on polymer or liquid crystal materials with conical anchoring on the interface in which the director tilt angle plays a key role in forming orientational structures, their response to external factors, optical parameters, etc.

## Data Availability

The data presented in this study are available on request from the corresponding author.

## Supplementary Materials

The following are available online at https://www.mdpi.com/article/10.3390/polym13172993/s1, Figure S1: Flow effect in LC cells with the one substrate covered with a mixture of PiBMA: PMMA without the addition of nematic LN-396; Figure S2: Azimuthal orientation of the director near the domain border and the “trace”, and violation of the director tilt angle at the domain border only.
