# Supplementary material for: Nematic Structures under Conical Anchoring at Various Director Tilt Angles Specified by Polymethacrylate Compositions
Source: Polymers (Basel). 2021 Sep 3;13(17):2993. doi: 10.3390/polym13172993 (PMC8434558; doi:10.3390/polym13172993)
Supplement: Supplementary file 1 [file polymers-13-02993-s001.zip › polymers-1347550-supplementary.pdf]

# Supplementary Materials: Nematic structures under conical anchoring at various director tilt angles specified by polymethacrylate compositions

**Denis A. Kostikov [1,2]*, Mikhail N. Krakhalev [1,2], Oxana O. Prishchepa [1] and Victor Ya. Zyryanov [1]**

## 1. Flow effect in LC cells with the one substrate covered by a mixture of PiBMA : PMMA without the addition of nematic LN-396

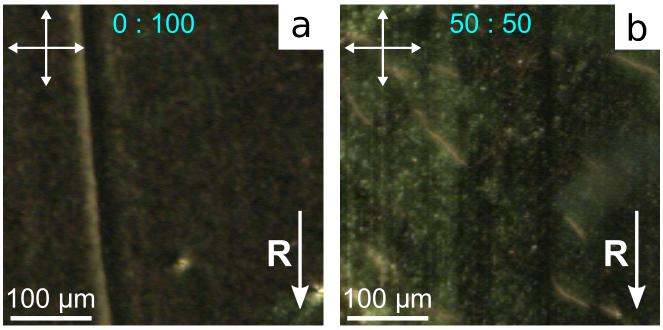


**Figure S1.** POM photos of LC cells with the bottom substrate covered by the rubbed PVA film and the top substrate covered by the film of polymer mixture PiBMA : PMMA = 0 : 100 (a) and 50 : 50 (b). The solid arrow indicates the rubbing direction **R**, the polarizer directions are indicated by double arrows.

## 2. Azimuthal orientation of the director near the domain border and the "trace", and violation of the director tilt angle at the domain border only.

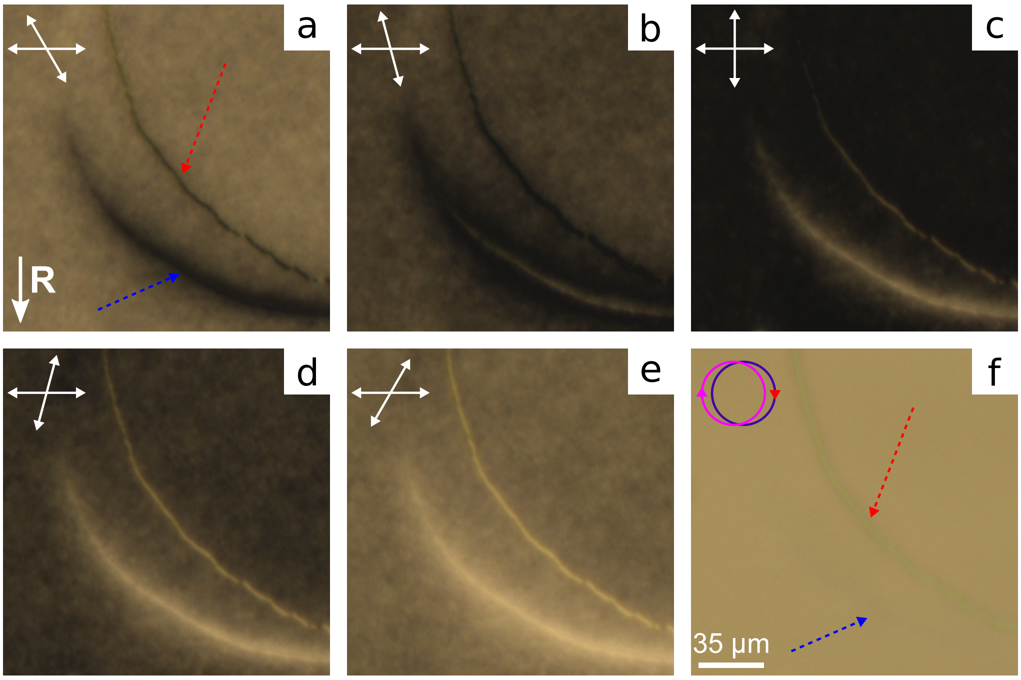


**Figure S2.** POM photos of LC cell with the bottom substrate covered by the rubbed PVA film and the top substrate covered by the film of PiBMA:PMMA: LN-396=80:20:20. Photos are taken at the angle between polarizer and analyzer $-30°$ (a), $-60°$ (b), $90°$ (c), $60°$ (d), $30°$ (e), and in crossed circular polarizers (f). The solid arrow indicates the rubbing direction **R**; the linear polarizer directions are indicated by double arrows; the circular polarizer directions are indicated by the circles. The domain border and "trace" are marked by red and blue dashed arrows, respectively.
